# How do medical journalists treat cancer-related issues?

**DOI:** 10.3332/ecancer.2015.502

**Published:** 2015-01-26

**Authors:** Haruka Nakada, Masaharu Tsubokura, Yukiko Kishi, Koichiro Yuji, Tomoko Matsumura, Masahiro Kami

**Affiliations:** Division of Social Communication System for Advanced Clinical Research, Institute of Medical Science, The University of Tokyo, 4-6-1 Shirokanedai, Minato-ku, Tokyo 108-8639, Japan

**Keywords:** information seeking, information source, medical information, news reporting, patient behaviour

## Abstract

Cancer patients can obtain information about their illness through a variety of media sources. Therefore, it is important to know how medical journalists treat cancer-related issues; to that end, we sent self-administered questionnaires to 364 journalists in 82 organisations who had reported on medical issues for the Japanese media, asking for their reasons for reporting on cancer-related issues and the difficulties they had faced. The most common reason for reporting on health-related issues was their personal interest in a particular issue (*n* = 36). They mainly covered conventional therapies (*n* = 33), healthcare policy (*n* = 30), new therapies (*n* = 25), and diagnosis (*n* = 25). All of the journalists that were surveyed experienced some difficulties in reporting health issues. Significant concerns included the quality of information (*n* = 36), social impact (*n* = 35), lack of technical knowledge (*n* = 35), and difficulty in understanding technical terms (*n* = 35). Journalists commonly used personal networks, including physicians, as information sources (*n* = 42), as well as social media (e.g., e-mail, Twitter and Facebook) (*n* = 32). Topic selection was biased, with 35 of 48 journalists having never reported on topics concerning hospices. Physicians were the most trusted source of information about cancer, and journalists attached high importance to interviewing them. As medical knowledge is advancing rapidly, journalists may have increasing difficulty covering cancer-related issues.

## Introduction

Cancer is a significant problem worldwide, including in developed countries. After diagnosis, cancer patients seek information that is accurate, accessible, and easily understood. They want to know which physicians they should choose, what kind of therapy they might receive, and how cancer will affect their lives [1]. Patients rely on a number of different sources of information, with primary care physicians being the number one source. Nagler *et al* (2010) found that the most frequently cited sources of information among cancer patients were doctors (75%) [2]. Most physicians give highly individualised advice to their patients according to the type of cancer and the patient’s medical history.

However, patients also rely on the media for information, including television, newspapers, and magazines. Approximately 260 cancer-related articles are published every year by the British Broadcasting Corporation (BBC) online [3]. In Japan, approximately 16,000 cancer-related articles—1.3% of total articles—are published every year in the five major newspapers [4]. The development of social media, including e-mail, Twitter and Facebook, has expanded the influence of the media on the public understanding of cancer [1].

Despite cancer being a common media topic in ageing societies, with most individuals obtaining cancer-related information through the media, little is known about how the media itself treats cancer-related issues. Therefore, we have investigated how medical journalists select topics, obtain information, and publish articles on cancer-related issues.

## Methods

We sent self-administered questionnaires via e-mail to 364 journalists in 82 organisations who had reported on medical issues in newspapers, television, radio, weekly magazines, or web-based media. We set a deadline for response, gave them a two-month period, and sent a reminder before the deadline. Sources included non-commercial and tabloid media as well as commercial and broadsheet media. All media companies belonging to the press club of the Ministry of Health, Labour, and Welfare were represented.

The questionnaire consisted of 24 multiple-choice and short-answer questions, which were classified into three groups: 1) demographic information (age, sex, educational status, and career); 2) difficulties faced in reporting on medical issues (14 topics included time and space constraints, relationships with sponsors, and difficulty understanding technical terms); and 3) reporting styles, including topics, information sources, and types of cancer discussed. The study conducted between December 2010 and January 2011 was approved by the Research Ethics Committee of the Institute of Medical Science, The University of Tokyo.

## Results

Fifty-seven of the 364 journalists contacted responded (16% response). Most companies belonging to the press club were represented in those journalists responding to the questionnaire. Of the 57 participants, 48 who had reported on cancer topics were enrolled in the study.

The backgrounds of the 48 participants were as follows. Their median age group was the 40s (range: 20s–60s). Of the participants, 46 (96%) had been educated beyond a university degree. Among them 34, 13, and 1 participants had majored in the humanities, natural science, and medicine, respectively.

Journalists chose to work on health-related issues based on personal interests (*n* = 36), personal experience as a patient (*n* = 4), illness of a family member (*n* = 6), and the influence of family members who worked as medical professionals (*n* = 3).

**Figure 1.**
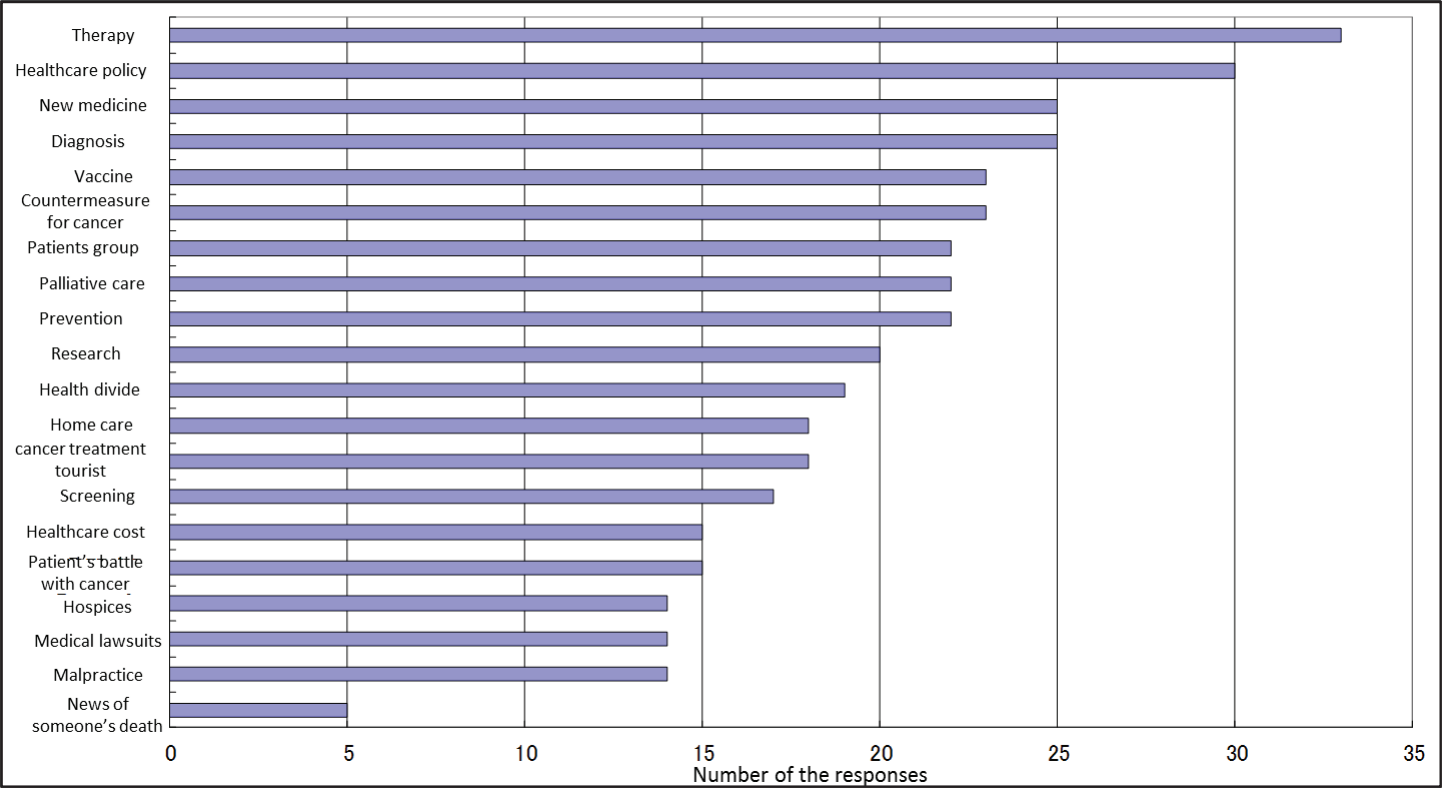
Topics relating to cancer on which the 48 journalists had reported.

**Figure 2.**
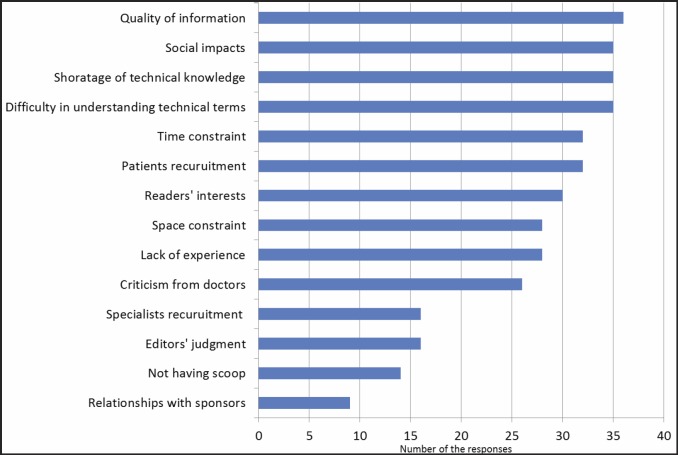
The journalists all had some difficulties in dealing with health care issues.

Most of the journalists (*n* = 33) had reported on cancer therapy (Figure 1). All journalists cited some difficulties in reporting on health-related issues (Figure 2).

**Figure 3.**
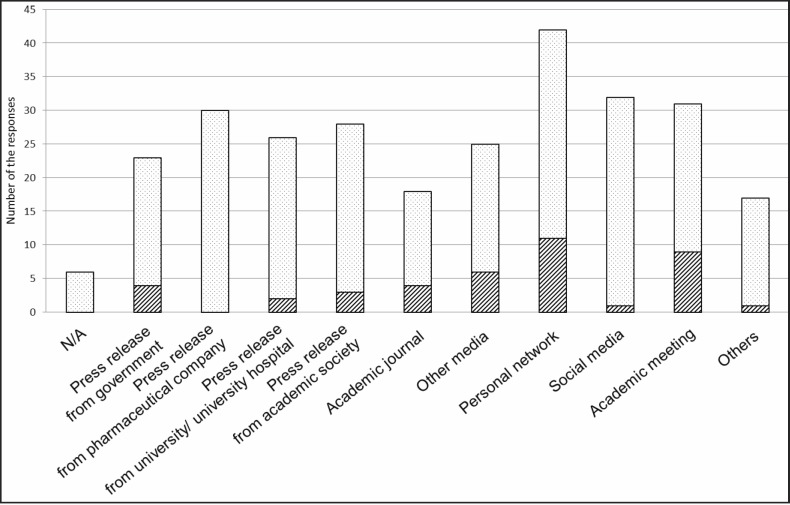
We asked the journalists to rank their information sources. They cited many information sources. Striped bars show the primary information sources and dotted bars show secondary ones.

Journalists used a wide variety of information sources, their personal network, including physicians, being their most frequently used source (*n* = 42). Social media were cited frequently (*n* = 32), but only one participant cited them as a primary information source. In contrast, 9 of the 48 journalists cited academic conferences as their primary source of information. None of them cited press releases from pharmaceutical companies as their primary source (Figure 3).

## Discussion

This study provides important information about journalists who publish articles on cancer. First, the selection of topics is clearly biased; for example, aggressive treatments and survival rates attracted the attention of journalists much more than treatment failure, adverse events, end-of-life care, and death. Unexpectedly, 35 of the 48 participants (73%) had never reported on hospices, which is comparable to previous findings that only 7.6% of cancer-related articles focused on death and dying, whereas 32% focused on survival [5]. This bias may give patients or the general public an inaccurate and optimistic view of the experience of cancer. The journalists should select the topics not for their interests, but for patient’s information needs. For physicians, on their part, they should provide appropriate information contributing to the treatment and end-of-life decision.

Even journalists with long careers shared difficulties in reporting on health-related issues. The most significant concern was for the quality of information, with 36 participants (73%) being concerned. The next major concern was social impact (*n* = 35). Considering that even physicians with advanced medical knowledge can be influenced by biased information [6], these concerns are reasonable. While journalists aim to write accurate reports, they face a number of obstacles, including difficulty in understanding technical terms (*n* = 35), difficulty recruiting patients for interviews (*n* = 32), and time constraints (*n* = 32). Medical knowledge is rapidly advancing and becoming increasingly specialised, making it increasingly difficult to provide accurate medical information to the general public.

To prevent biased reporting, journalists should employ a broad range of information sources. However, we found that 42 of the 48 journalists (88%) depended mainly on their personal network for information, as in previous studies [7]. Despite newly available communication tools, physicians were the most trusted source of information about cancer, and journalists attached great importance to interviewing them.

The development of social media has made it increasingly easy to gather information through personal networks [8]. Only one journalist, however, cited social media as a primary information source, whereas 32 of the 48 journalists (67%) used social media to glean information for their articles. As anyone can post healthcare information through social media, regardless of their credentials or lack thereof [9], journalists may understandably be skeptical about the reliability of social media information.

Journalists did not cite academic journals as a frequent source of information, with only 18 of the 48 journalists (38%) listing them in the present study. Most physicians hope to publish their research in academic journals, but these are clearly ineffective in communicating medical information to journalists, which may be due to difficulty understanding technical terms and jargon. Alternatively, journalists may not be seeking the highly specialised level of discussion characteristic of academic journals. They would rather focus on new information and developments that they deem useful for their readers [10], and academic journals simply do not fit their needs.

As expected, no journalist cited press releases from pharmaceutical companies as the primary information source. As this information is frequently highly biased [11] it is not usually an appropriate source of news for the public. Journalists ought to pay attention to the authors of press releases to check any potential conflict of interest in conveying accurate information.

Most journalists were sensitive to the potential social impact of their articles; however, 14 journalists (29%) cited not having a scoop, an important news report reported first by one organisation, as a difficulty in dealing with healthcare-related issues. Furthermore, 20 of the 48 journalists (42%) had reported on medical lawsuits or malpractice suits associated with cancer. In general, journalists prefer to report sensational events rather than institutional issues in medical care. These tendencies may increase the risk of public misunderstanding of healthcare-related issues.

The gefitinib scandal (Iressa^TM^, AstraZeneca PLC, UK) is typical of the sort of cases reported in Japan [12]. In this scandal, several media outlets reported the drug to be highly effective in certain types of lung cancer, without significant adverse events, leading many physicians and patients to consider it a ‘dream drug’ [13]. Later, a number of patients taking gefitinib died of its adverse events. Media reporting on this ‘miracle’ drug may have exacerbated the disaster by raising its public profile and encouraging people to consider it less dangerous than it actually was.

This study provides valuable insight into the approaches of journalists who report on cancer-related topics, but this study has some limitations. First, the sample size was small; there might be a bias in which journalists responded. Because of the small sample size, the result might not represent the attitude of Japanese media.

Second, the uptake was low (16%). This may be because some news outlets selected only one respondent to represent the whole organisation. Due to the low response, the results might be biased towards journalists with more liberal opinions, and therefore larger sampling would be required to verify or refute the findings.

Third, the detail of the approaches of journalists dealing health care issues was not identified. To analyse their approaches toward reporting cancer-related issues in more detail, a qualitative study would be needed.

## Grant Support

This study was supported by a Grant-in-Aid from the Ministry of Health, Welfare and Labor of the Japanese Government (Third Term Comprehensive Control Research for Cancer).

## Conflicts of interest

The authors declare that they have no conflicts of interest.
